# The burden of knowing: balancing benefits and barriers in HIV testing decisions. a qualitative study from Zambia

**DOI:** 10.1186/1472-6963-12-2

**Published:** 2012-01-05

**Authors:** Marte Jürgensen, Mary Tuba, Knut Fylkesnes, Astrid Blystad

**Affiliations:** 1 Centre for International Health, University of Bergen, Bergen, Norway; 2 Department of Community Medicine, School of Medicine, University of Zambia, Lusaka, Zambia; 3 Department of Public Health and Primary Health Care, University of Bergen, Bergen, Norway

## Abstract

**Background:**

Client-initiated HIV counselling and testing has been scaled up in many African countries, in the form of voluntary counselling and testing (VCT). Test rates have remained low, with HIV-related stigma being an important barrier to HIV testing. This study explored HIV testing decisions in one rural and one urban district in Zambia with high HIV prevalence and available antiretroviral treatment.

**Methods:**

Data were collected through 17 in-depth interviews and two focus group discussions with individuals and 10 in-depth interviews with counsellors. Interpretive description methodology was employed to analyse the data.

**Results:**

'To know your status' was found to be a highly charged concept yielding strong barriers against HIV testing. VCT was perceived as a diagnostic device and a gateway to treatment for the severely ill. Known benefits of prevention and early treatment were outweighed by a perceived burden of knowing your HIV status related to stigma and fear. The manner in which the VCT services were organised added to this burden.

**Conclusions:**

This study draws on social stigma theory to enhance the understanding of the continuity of HIV related stigma in the presence of ART, and argues that the burden of knowing an HIV status and the related reluctance to get HIV tested can be understood both as a form of label-avoidance and as strong expressions of the still powerful embodied memories of suffering and death among non-curable AIDS patients over the last decades. Hope lies in the emerging signs of a reduction in HIV related stigma experienced by those who had been tested for HIV. Further research into innovative HIV testing service designs that do not add to the burden of knowing is needed.

## Background

"I went there [the VCT centre] and even reached the door, and immediately I felt weak. I was scared and left. What if I was told I was positive? I would have died."

Woman, not accessed VCT

HIV counselling and testing (HCT) has been promoted as essential in working towards universal access to prevention, treatment, care and support [1,2]. Although client-initiated HCT has been scaled up in many African countries, demand is often low, despite studies demonstrating a high willingness to be tested [3-5]. The substantial gap between stated willingness to be tested and actually being tested indicates the presence of strong acceptability barriers against client-initiated HCT in its current form. A growing body of literature concerning HIV testing demonstrates how HIV test rates are shaped by diverse factors. Educational attainment appears to facilitate HIV testing [6-8], whereas risk behaviour presents a more unclear picture with higher test rates among individuals exhibiting high-risk behaviour in some areas [8] but lower test rates in others [9]. Stigma has been demonstrated to be an important barrier to HIV testing [3,10-13]. It also appears to influence testing indirectly through barriers related to confidentiality and privacy concerns [12,14]. Gender-specific barriers among women [5,15] are linked, in part, directly or indirectly to stigma. In some areas where access to antiretroviral treatment (ART) has been scaled up, reduced stigma and increased test rates have been demonstrated [16]. However, in other areas the emergence of new sources of stigma has counteracted this development [17], and in high-income countries stigma persists despite almost universal access to treatment [18]. Therefore, HIV-related stigma is located at the core of attempting to understand the low test rates in HCT programs.

HIV testing approaches have been much debated. The traditional approach has been client-initiated HCT, in the form of voluntary counselling and testing (VCT). A fundamental premise on which VCT is based is that everyone has the right to be tested and to know their HIV status [19]. It follows from this premise that the right not to know your HIV status is as fundamental as the right to know. Critics have argued that this rights-based approach has led to an AIDS exceptionalism, abandoning regular public health measures in prevention, and that this paradoxically has led to increased vulnerability and enhanced stigma [20]. Following these debates and in an attempt to increase HIV test rates, UNAIDS and WHO have recommended the implementation of provider-initiated testing in all health facilities of countries with generalized HIV epidemics [21]. This strategy implies that HCT is incorporated into routine medical care. It is thought that this will diminish the effects of stigma, as HIV testing will be perceived as an integral part of the general health assessment. Within a provider-initiated HCT strategy pre-test counselling is simplified compared with VCT, and it is commonly characterized by an opt-out approach, implying that clients who do not wish to be tested will actively have to state a lack of willingness to be tested. As provider-initiated testing has been scaled up, primarily as part of the prevention of mother to child transmission (PMTCT) programmes, but also increasingly in general health services, concerns have been raised concerning the quality of counselling and particularly the consent process, as clients may experience limited or no possibility of opting out and may perceive HIV testing as mandatory [22-24].

### The stigma concept

Stigma has been a component of the HIV/AIDS scenario since the onset of the pandemic, and a large body of research exists concerning diverse aspects of the phenomenon. The first associations between stigma and health in social science literature date back to the 1880s, but the stigma concept was first fully introduced in the classical sociological works of Goffman, who defined stigma as "an attribute that is significantly discrediting" [25]. According to Goffman, the stigmatized person possesses an "undesirable difference", and he saw the conceptualization of what constitutes this 'difference' or 'deviance' as a social process. Goffman's idea of stigma as an attribute has strongly influenced later authors, leading to highly individualized analyses of stigma, particularly within social psychology [18]. Although they have developed substantial knowledge, social psychological works have been criticized for having too strong a focus on the individual and for placing little emphasis on the social and structural aspects of stigma [16,18,26-28].

Link and Phelan introduced a more sociologically oriented approach to understanding stigma, aiming to link individual and social stigma components and to emphasize stigma as a constantly changing social process [28]. In their conceptualization, stigma exists when five inter-related components converge; namely 'labelling', 'stereotyping', 'separation', 'status loss and discrimination', and the playing-out of 'social and political power'. Discrimination can be individual, structural or self-imposed [18,28]. Parker and Aggleton have added that stigma and stigmatization occur not merely as a reaction to differences, but in relation to social and structural inequalities, and that "stigma plays a key role in producing and reproducing relations of power and control" [26]. Castro and Farmer further argue that structural violence, the large-scale social forces that shape every society, including social inequalities and poverty, to a great extent shape stigma and discrimination and determine who suffers from it [16].

Stigma research has been criticized for neglecting the lived experience of those affected by it [27,28]. Anthropologists have launched meaning centred approaches, arguing that the stigma concept remains empty and decontextualized if not filled with meaning from people's lived experiences [16,29]. They suggest seeing stigma as a fundamental moral experience, as stigmatized conditions threaten what is most at stake for sufferers. Therefore, the process of stigmatization is a pragmatic response to "perceived threats, real dangers, and fear of the unknown" [27]. Approaching stigma as a moral experience allows us to understand those stigmatizing and those being stigmatized, as both are involved in interpreting, living out and reacting to what matters most and what is threatened. For analytical purposes, stigma can be divided into enacted stigma and felt stigma [30]. Enacted stigma can be seen as stigmatizing attitudes and acts of discriminations by the public, whereas felt stigma is the stigmatized person's internal feelings of shame (self-stigma) and fear of discrimination (perceived stigma).

Drawing particularly on the writings of Kleinman and Link and Phelan, this study pursues the central question of interpreting what is at stake for particular persons in a particular context at a particular time. In line with meaning centred approaches [31], this work argues that assessing the particularities of the manner in which the substantial continued reluctance towards testing for HIV was expressed and where treatment was available is of substantial value in understanding what is at stake in an HIV testing context.

### HIV prevalence and HIV testing in Zambia

Adult HIV prevalence in Zambia has been estimated at 13.5% [32], and during the last decade important shifts in the epidemic have been recorded. A decline in HIV prevalence among young people aged 15 - 24 years has been documented, probably reflecting a reduction in incidence in this age group [33,34]. A marked shift in the association between educational attainment and HIV infection to reduced risk of HIV infection in more educated groups, particularly among young people [35], has been explained by reduced risk behaviour in these groups [36]. However, in some areas the prevalence has increased [37], revealing a complex situation with diverse dynamics.

Client-initiated HCT has been available in Zambia since the late 1980s in the form of VCT. There was limited geographical coverage to begin with, but since 1998 there has been a country-wide rollout. In 2007 there were approximately 700 government- and NGO-run VCT sites in Zambia (Bristol Cheembo 2007, personal communication). At the time of this study, provider-initiated testing was applied in PMTCT-programmes, but not in the general health service. In 2007, 35% of women and 20% of men had been tested for HIV and received the results [38]. This study explored local meanings related to client-initiated HCT in a rural and an urban setting in Zambia, with the aim of increasing knowledge concerning obstacles to HIV testing in settings with a long established high prevalence of HIV and available antiretroviral treatment. Drawing on social stigma theory, the aim of this study was to increase understanding of the continuity of HIV-related stigma in such settings, and to generate knowledge that could improve HIV testing strategies.

## Methods

The present study was conducted in 2007 in two districts of Zambia: Kapiri Mposhi is a predominantly rural district located in the Central Province with a population density of 10.7 per sq km, and Lusaka is an urban province with a population density of 63.5 per sq km [39]. In 2005, VCT was available in 58% and antiretroviral treatment (ART) in 9% of health care facilities in the Central Province, whereas in Lusaka VCT was available in 52% and ART available in 32% of health care facilities [40]. However, at the time of the study ART was being scaled up, and the availability of these services was expected to be higher than in 2005. In Central Province 15.0% of men and 16.1% of women had been tested for HIV and received the result in the year prior to the survey, whereas in Lusaka Province the corresponding figures were 13.6% and 24.8% [38].

To explore the obstacles to VCT, a qualitative study design with in-depth interviews and focus group discussions was chosen. The focus group discussions were not part of the original research protocol but were included to enrich the data, to validate the findings from the interviews and to ensure that young people were represented. We expected that individual interviews would be most appropriate for the data collection, due to the sensitive nature of HIV testing decisions, and was therefore surprised by how much the focus group discussions added to and enriched the material.

In total, 17 in-depth interviews and two focus group discussions were conducted with individuals (Table 1), and 10 in-depth interviews were conducted with VCT counsellors (Table 2). Individuals were recruited on a voluntary basis, with the assistance of neighbourhood health committees or health workers. Purposive sampling was employed to ensure the participation of informants who had accessed VCT and informants who had not used the service, and to ensure variation in terms of age and gender. The informants are described in table 1. We decided to include both men and women in the same focus groups, because HIV testing experiences were seen as a cross-cutting issue and because we considered it to be appropriate and acceptable in this setting, based on previous experience in the same areas. The active discussions, with both men and women participating actively, confirmed this. Counsellors were purposively sampled from various service providers to include a diversity of experiences. Three counsellors were recruited from public hospital-based or clinic-based VCT centres and five from three NGOs providing VCT, and two were community counsellors. The counsellors interviewed are described in table 2. Recruitment of further informants (individuals and counsellors) was ended when we got a sense of saturation through observing major recurrent patterns with regards to testing in the data material.

**Table 1:** Overview of informants

Location		Person	VCT status	Sex	Age	Comment
Kapiri	IDIs	1	Accessed	Female	36	
		2	Accessed twice	Female	50	
		3	Accessed	Male	49	
		4	Accessed	Female	34	
		5	Not accessed	Female	46	
		6	Not accessed	Male	54	
		7	Not accessed	Male	28	
		8	Not accessed	Female		Not transcribed
	
	FGD	1	Not accessed	Male	18	
		2	Not accessed	Male	32	
		3	Not accessed	Female	49	
		4	Not accessed	Female	20	
		5	Not accessed	Female	36	
		6	Not accessed	Female	35	
		7	Not accessed	Female	19	
		8	Not accessed	Male	17	
		9	Not accessed	Male	20	

Lusaka	IDIs	1	Accessed	Male	50	
		2	Accessed	Female	32	
		3	Accessed	Male	35	
		4	Accessed	Female	40	
		5	Accessed	Male	42	
		6	Accessed	Male		Not transcribed
		7	Not accessed	Male	33	
		8	Not accessed	Female	26	
		9	Not accessed	Female	38	
	
	FGD	1	Not accessed	Male	22	
		2	Not accessed	Female	18	
		3	Not accessed	Female	22	
		4	Not accessed	Male	23	
		5	Not accessed	Female	21	
		6	Not accessed	Female	24	
		7	Not accessed	Male	24	
		8	Not accessed	Female	18	

**Table 2:** Overview of counselors interviewed

Counsellors		Service type	Sex	Comment
Kapiri	1	Hospital	Female	
	2	NGO	Male	
	3	NGO	Male	
	4	Community	Male	
	5	Community	Male	

Lusaka	1	NGO	Male	
	2	NGO	Female	Not transcribed
	3	NGO/clinic	Male	
	4	Clinic	Female	
	5	Clinic	Female	

The in-depth interviews were guided by semi-structured interview guides drafted prior to the study. The guides were revised and further developed throughout the interview process, and questions concerning barriers to HIV testing were added after the emergence of these topics during earlier interviews. The focus group discussions were guided by a topic guide, covering the same major topics as the interview guide. As the focus group discussion was conducted at the end of the data collection in each study area, it also incorporated information obtained in the individual interviews. The interviews and focus group discussions were carried out in people's homes, outside or at health facilities, depending on convenience and the preference of the participants. The focus groups and the majority of individual interviews were conducted in the local language by a medical anthropologist (the second author) or a trained research assistant with substantial experience from qualitative work. Some interviews were conducted in English by the first author, including the interviews with counsellors. The interviews were recorded, transcribed verbatim and translated directly by the research assistant. The aim was to retain transcripts verbatim and translations as close as possible to the actual stated content. Three interviews were excluded due to poor sound quality in the recordings; one counsellor, one individual who had accessed VCT and one individual who had not accessed VCT. The early interviews were followed by a thorough discussion of the content within the research team, as part of the continuous reflection and analysis. Informal discussions with health personnel, informants before and after interviews, and others informed the analysis.

Drawing on interpretive description methodology, the data were processed and analysed throughout the research process [41,42]. Conceptual themes were derived inductively from reading and discussing the early transcripts, through constant comparison within and between the interviews, searching for both commonalities and differences. The key themes identified in each interview were further explored systematically and challenged in succeeding interviews and focus group discussions, and subsequently in the more rigorous processes of analysis by the first author after collection of the data was completed. As the in-depth interviews and the focus group discussions covered the same topics, it was natural to integrate both in the analysis. Hence, the in-depth interviews and the focus group discussions were treated equally in the analysis, with comparison both within and between the transcripts. The major message of VCT campaigns, "to know your status", emerged as an overarching category in the analysis. This message had diverse meanings to the informants, and these meanings emerged as themes. To know your status was seen as a benefit, such as taking care of your health, gaining access to treatment and preventing transmission of HIV. However, to know your status could also mean to know you would be discriminated against, fear of moral judgement, fear of loss of future opportunities, and an experience of getting a death sentence. These meanings are described in the Results section.

All participants provided oral consent for participation in the study. No names were recorded, and confidentiality was ensured throughout the interviews and focus group discussions. The study was approved by the Research Ethics Committee at the University of Zambia. In both districts, permission was obtained to work with clinic staff and neighbourhood health committees from the District Medical Officer and the local health facilities. Permission to interview staff was obtained from NGOs providing VCT within the study districts.

## Results

### Perceived benefits of VCT

Two major benefits of VCT were continuously brought up during the interviews and focus group discussions in both research settings, namely receiving a diagnosis related to deteriorating health and receiving access to treatment. Of the ten informants who had accessed VCT, eight had done so because they had experienced health problems and wished to confirm their suspicion of being HIV-positive. Several of the informants had been so severely ill that they in reality had not made the decision to be tested themselves.

"Before I went for VCT, I was very sick most of the time. Malaria never ended, I was feverish and had sores in my mouth. That's when I thought of going for VCT."

Woman 36 years, accessed VCT, IDI

For these informants, VCT was employed as a diagnostic device after they had fallen ill. Although the informants who had accessed VCT had knowledge of the potential preventive benefits of VCT and emphasized the importance of being tested while one was healthy, only two of the informants were healthy when they accessed VCT. One of these was tested because she considered herself to be at risk owing to her work as a traditional birth attendant, whereas the second was the only informant to be tested simply to know his HIV status. The following quotations from one of the informants are illustrative of the apparent contrast between knowing the importance of testing and actual behaviour. When asked when people should generally go for VCT he replied:

"Any time, whenever they feel they have to know their status...one should go for it."

However, when asked about why he had thought of going for VCT himself he said:

"I started suspecting that there might be something wrong with me. My weight started decreasing, then I got a lot of diseases that I had never had before. So with me I can say I was lucky because that made me go for it [VCT]."

Man, 50 years, accessed VCT, IDI

Similar reasoning became evident in response to the question of whether one should be tested if treatment was not available. While several informants who had been tested argued for the importance of knowing one's status even if treatment was not available, most informants said that they would not have been tested if there was no treatment. The counsellors confirmed that the majority of their clients came for VCT because of illness and with the aim of starting treatment.

Respondent: Most of the people who decide to come for VCT; it's because of the ART clinic.

Interviewer: So if there was no medicine...?

Respondent: It wouldn't happen. People would not come for VCT.

Man 33 years, not accessed VCT, volunteer at ART clinic, IDI

The apparent ambiguity of knowing the benefits of testing, combined with a reluctance to get tested while still healthy, was evident among informants who had not accessed VCT. Most of these informants eagerly emphasized the health benefits of counselling and of being tested for HIV while still being well and healthy. Some of the informants also stated they were motivated to be tested, highlighting these benefits. Simultaneously, they expressed that for themselves they would not seek VCT before they fell ill and needed treatment. The following brief dialogue provides an illustration of the apparent ambivalence present in the informants' accounts:

I: So why are you saying VCT is a good thing?

R: Because I have seen it has helped a lot of people. Those found to be positive, now they are being given the ARVs. So I think it's good. (...) I think for someone who is negative, it is good because such a person will continue protecting his or her life. (...)

I will go there one day but not now.

I: Not now?

R: Not now, because I am not ready.

I: What is making you not to be ready?

R: Mmm...I am scared.

I: You are scared of what?

R: Of finding myself to be positive. I don't know how I will live. I am scared to be told that I am HIV-positive. I think I will die. (...) Now what I think is when I get sick I will go for VCT.

I: So you can only go for VCT when you are sick?

R: That's what I think.

Woman, 26 years, not accessed VCT, IDI

One of the informants interviewed worked as a volunteer at the local ART clinic. When asked about when he would go for testing he said:

R: One day I will think of doing so.

I: When...?

R: When I am ready. (...) You see, if a person is healthy, and he is not feeling anything, there is no need for going for VCT.

I: Do you think so?

R: (laughs) This is what I think.

I: But I asked you a question earlier about when one should go for VCT and you said 'anytime'.

R: Yeah, it depends, but you see most of the people go for VCT when they are sick.

Man, volunteer at ART clinic, 33 years, not accessed VCT, IDI

Even VCT counsellors did not always appreciate the preventive benefit of accessing VCT while feeling healthy:

"Actually, we do talk to them about prevention, but . . . actually, what I am trying to say is that we do talk to them about prevention. But if a client comes here, the client needs to have treatment."

Counsellor, NGO, IDI

### Barriers to VCT - The burden of knowing

HIV testing decisions were characterized by a disturbed assessment of benefits and barriers, and although informants were aware of the preventive and treatment benefits implied in testing, strong barriers held them back from being tested. It emerged from the interviews and focus group discussions that 'knowing your status' was experienced as a highly charged concept filled with meaning from the informants' long term experiences with HIV/AIDS. Deep fears regarding HIV testing were revealed, which emerged in surprisingly similar ways in both research settings, and across age groups and gender.

When asked why they would not go for VCT, informants reported a general fear of knowing their status, although they knew that people could live for long periods with treatment. Several suspected that they could be HIV-positive because of previous risk behaviour, but the diagnosis frightened them so much that they would rather not know. Informants held that the worry in itself would accelerate the disease and that they would die faster if they knew they were HIV-positive, as revealed below:

"I don't know if I am positive or negative. It's better I am just like this because I look fit. But when I know my status I will have mental torture, I will get thin. And I will only live a few years and die early. Then what have you done on earth? Nothing."

Man 24 years, not accessed VCT, FGD

"Now what I think is when I get sick I will go for VCT. Because if I go for VCT now and the result is [HIV] positive, then I will get sick because I will be thinking a lot."

Woman 26 years, not accessed VCT, IDI

For many informants the prospect of intense thinking and worrying was perceived as being so detrimental to their health that they would rather not know. Although treatment was available in both study areas, those who had not been tested still talked about HIV as a death sentence, as was confirmed by the counsellors:

"What is it that they fear? Just HIV in itself. Because they think that once you are HIV positive, the next thing is death."

Counsellor, NGO, IDI

In the rural study area, several informants told anecdotes about people who had committed suicide after testing positive for HIV in order to avoid the immense social and physical suffering they had seen among those dying from AIDS.

"There are people who commit suicide immediately after they have been told that he or she is HIV positive. In this area there have been five people. Young men, mostly. One of them threw himself in a pit and died."

Woman 34 years, accessed VCT, IDI

HIV-positive people were often labelled as 'moving corpses'. Another common label was the Nyanja term '*kanayaka*': literally translated as '*it has ignited' *or '*the fire has started*'. This expression is commonly used for contagious diseases, but the interpretation has gained additional dimensions in an AIDS context with the interpretation being that one's life had come to an end, and that the inevitable course against death could not be prevented. In addition, the notion of fire pointed to a warning light, making a person's risk behaviour and HIV status visible, and signalling that death had come or one was already dead. It also indicated that the person posed a danger to the community, and igniting the fire was thus a metaphor for the suspected spread of the disease to other people, like the spread of fire.

"They laugh at you if you go for VCT. It means that you have that suspicion of being HIV positive. They place you as already positive if you go for VCT. They say: Look at that one, she is even going for VCT, maybe *kanayaka*!"

Woman 32 years, accessed VCT, IDI

"So this is the reason why some of us...we fear to go for VCT. People will say *kanayaka*, he will die soon."

Man 33 years, not accessed VCT, IDI

As the most common motivations for HIV testing were accelerated deterioration of one's health or a perceived risk, it was common to assume that those presenting for testing were already suffering from AIDS and that they had 'misbehaved' sexually. Although many informants, particularly counsellors, would state that the HIV related stigma was less evident than in the past, HIV was still, after several decades, strongly associated with promiscuity and prostitution, particularly among those who had not been tested. Most of the informants linked an HIV diagnosis, or just being seen at the VCT site, with substantial loss of moral standing and discrimination from families and communities.

"The fear that I know, it's the way people are going to look at you. Maybe everyone in the community they know me as a person. They respect me today, but the moment they hear I am HIV positive, they conclude: 'so she has been sleeping around. She was just pretending to be a good person at home or in the community'."

Woman 24 years, not accessed VCT, FGD

Young informants expressed strong barriers to VCT. They articulated concerns about the loss of future opportunities for education, work and marriage if they were tested and found positive, both due to the loss of moral standing and due to the assumption that they would die soon. Several informants stated that just being seen at the VCT centre could mean the end of such future opportunities.

"Another reason why people don't want to go for VCT is, for example, at my workplace they don't want people with AIDS. If I they found out that I have the virus, it means that I will stop working there and then . . . so for me, even if I get sick I can't go for VCT. So that I lose my work? No."

Woman 22 years, not accessed VCT, FGD

"Like me, I am a student. So I wouldn't feel good if I was found to be HIV positive, because I would think why should I get educated? Very soon I am dying. It's even better to stop [education]."

Woman 18 years, not accessed VCT, FGD

The fear of being seen as a living corpse, as a person on fire, and as a person with immoral conduct naturally had implications for perceptions of VCT centres.

"On the other side [of the clinic] people with malaria or other diseases wait, and when you come for VCT you must pass them. And when they see you, people say 'obviously that one misbehaved, why else is she going there?"

Woman 24 years, not accessed VCT, FGD

The fact that in many cases the arrangement of the VCT facilities compromised confidentiality and contributed to the 'burden of knowing' was a striking finding. Usually VCT was located in a separate building or a particular room of the clinic, making it clearly visible to people. Informants feared being seen at the VCT centre or in the queue for VCT, as people would assume them to be HIV-positive and to have 'misbehaved'.

"Immediately, the moment they know that you have been for VCT, that's it. They start pointing at you saying that you have got AIDS. So that's why some fear to go for VCT, because everyone would think you have AIDS, even if you don't have. Just because you went for VCT you have AIDS. [...] Even when you just go to the hospital, they may say you have AIDS."

Man 54 years, not accessed VCT, IDI

"They will say we saw you or we saw him at the VCT centre. For example if you want to propose someone for marriage, it won't work if you've been seen at the VCT centre."

Man 32 years, not accessed VCT, FGD

Informants were worried about being seen at or leaving the VCT centre after testing and about how they would manage to keep a straight face in front of all the people at the clinic. In some clinics, HIV-positive patients still received a clinic card or envelope of a particular colour, different from the other clinic cards, making it readily visible to others who were the HIV patients.

"It's too open. You are just seated there [the clinic], and whoever you see with a khaki coloured envelope, you conclude they are on ARVs".

Woman 36 years, not accessed VCT, FGD

Some informants who had accessed VCT confirmed the concerns of stigma and discrimination. However, the picture was not unequivocal. A few of the informants who were HIV positive had not experienced discrimination in their communities, and held that their openness and improvement on treatment had encouraged others to go for testing.

"In the community there is actually no discrimination against me. I have even encouraged some people to go for VCT, and they have accepted the advice that I have given them."

Man 35 years, HIV positive, accessed VCT, IDI

## Discussion

The findings of this study demonstrate that VCT was not employed as a preventive measure, as stated as one of the aims of the service, but rather as a diagnostic tool after a person had fallen ill. To the majority of informants, the concept 'to know your status' meant confirming an expected diagnosis and gaining access to ART. Therefore, VCT was perceived as a diagnostic device and a gateway to treatment. The preventive messages concerning gaining knowledge about an HIV negative status and how to remain negative, or alternatively gaining knowledge about an HIV positive status and early access to treatment, were not perceived as powerful enough benefits to motivate people to go for testing. The fear of knowing ones HIV status originated from perceiving an HIV-positive result as a death sentence and from the anticipation of severe stigma. To the study participants, 'to know your status' meant to know that they existed somewhere between life and death or were already dead. 'To know your status' implied knowing that the fire had ignited and that life would inevitably soon burn out. Furthermore, an HIV positive status implied loss of moral standing, loss of support and loss of future opportunities. It meant living with a warning light flashing for everyone to see and talk about. In this manner 'to know your status' conjured up meanings so strong that the burden of knowing outweighed the benefits of prevention, as well as of early treatment.

Briefly drawing on the writings of Link and Phelan [28] concerning stigma processes may shed light on the complex processes at work. As described in the background section, the authors suggest five inter-related components of stigma, namely: (i) people distinguish and label human differences (labelling); (ii) dominant cultural beliefs link labelled persons to undesirable characteristics (stereotyping); (iii) labelled persons are placed in distinct categories separating 'us' from 'them' (separation); (iv) labelled persons experience loss of position and are commonly discriminated against in ways that lead to unequal outcomes (status loss and discrimination); (v) stigmatization is contingent on access to ways of playing out social, economic and political power. In the current study, people were labelled and pointed out as HIV positive, sometimes just by being seen at the testing site. Stereotyping surfaced by linking an HIV positive status to the 'immoral' characteristics of promiscuity and prostitution, and even more strongly through the concepts of 'moving corpses', 'living dead' and 'fire being ignited', suggesting that HIV is seen as an unnatural state threatening the usual categories of life and death [43]. These labels and stereotypes were employed to mark a distance between 'us' and 'them' actively. In order to distinguish between right and wrong people will everywhere mark a separation between moral and immoral realms or communities, and people may go to great lengths to ensure that they remain within the right category. A separation is thus created between moral and immoral conduct, and in our study HIV positive people were located in an immoral category that was separate from the normal moral community. Being located in the immoral category may have devastating implications for one's reputation and in terms of loss of status, leading to discrimination and marginalisation. For example, the perceptions of HIV positive people as 'dead' or 'living dead' had implications for future investment in people in terms of education, jobs and marriage. Not only did an HIV positive status imply a threat to an individual's future opportunities, but it also implied a threat to the people around him or her. The metaphor of igniting a fire powerfully reveals the perceptions of the risk that HIV positive people are perceived to pose to the community, making it justifiable and possibly even important to label, stereotype and separate the infected from the non-infected. Discrimination also works to frighten others from gaining an HIV positive status. These findings demonstrate that gaining an HIV positive diagnosis was related to anticipation of stereotyping, status loss and discrimination, or a 'discredited status' to use Goffman's terms [25], to a point where the burden of knowing was perceived as too heavy to relate to. The process of stigmatization of HIV positive individuals implies the playing out of power gained from the sense of being part of a moral community, being clean, healthy and alive vs. a community of immoral, dead or dying individuals that one should fear. This power dimension has been noted by diverse scholars of HIV [16,26], but has not been explored in depth in the present study.

Drawing on stigma research from other fields within health may be useful when attempting to understand how HIV-related stigma influences HIV testing seeking behaviour. The modified labelling theory has been suggested to explain how labels may lead to negative outcomes in individuals [44]. People may be labelled as HIV-positive in different ways. Labels may be obtained from others, such as receiving a diagnosis from a doctor or a counsellor, or they can be acquired by association [45], such as being observed at the HIV testing room or centre. Public labelling by association, such as being seen at the VCT centre or room, was common in the two research settings. The fear of enacted stigma after being labelled by association is prominent among our informants, and their decision to avoid testing as long as possible can therefore be seen as a form of label-avoidance. By avoiding the test rooms and clinics, they avoid being labelled by the community and thereby avoid the anticipated public stigma and its harm. In our material we also see how self-stigma is prominent. In an area with a longstanding epidemic and widespread stigma, people will tend to internalize the stigmatizing attitudes that they have experienced as common in the community. People at risk of being HIV infected will therefore think that they are less worth if they are found to be HIV positive. This may lead to low self-esteem and low self-efficacy [28,45], i.e. what we call self-stigma. The poor self-efficacy is particularly evident among the young informants in our study, who believed they would lose future opportunities if found HIV-positive. In our material we can understand the intense fear and worrying as a strong expression of self-stigma. Many of the informants actually believed they would die of worry if they were to be labelled as HIV-positive, which naturally held them back from being tested for HIV. This can thus also be seen as a form of label avoidance. By not knowing their 'label', and not seeking to know it, they could avoid the fear, shame, devaluation of themselves and the low expectations to future opportunities.

Yang et al. talk of stigma as a moral experience, seeing stigmatized conditions as threatening what is most at stake [27]. Also in the context of ART availability, an HIV positive diagnosis continues to threaten what is most at stake for people: moral standing, prospects of a spouse and family, access to education and work, good health and survival. Such devastating threats to what is at stake in local moral worlds have created a fear so strong that an HIV positive diagnosis was assumed by our informants to lead to worries causing accelerated disease and an early death, as has also been found in studies carried out in other African settings [12,46,47]. A population survey in Zambia demonstrated that a substantial part of the mental distress related to HIV was mediated through self-perceived risk and worry of being HIV infected [48].

It has been anticipated that long experience of HIV in high prevalence settings and increasing availability of ART would cause stigma to gradually lose its power. In some settings such a decrease in stigma has been reported [16]. However, as is the case in other studies [17,49], this work reveals that stigma is still powerful and has a substantial impact on people's testing behaviour. Such continuity of HIV related stigma can only be understood if one attempts to grasp the implications of almost three decades of experience with HIV/AIDS implying early death and dying among the most productive and reproductive groups, reinforced by the long-standing message that this disease cannot be cured [43]. The memories of AIDS are embedded in narratives and experiences of death of people who under normal circumstances should not die, and have been powerfully imprinted in people's minds and bodies. These embodied memories [50] of an incurable disease associated with dehumanizing symptoms will affect future existential decisions including whether or not to be tested for HIV. As HIV comes with so much 'extraordinary baggage' the transition to a manageable chronic disease will be difficult and take time [46]. This study argues that the burden of knowing an HIV status and the related reluctance to get HIV tested are strong expressions of the still powerful embodied memories of the suffering experienced among non-curable AIDS patients.

This study indicates that the way in which VCT services were organised substantively added to the burden of knowing one's HIV status. The informants revealed that being exposed at VCT sites was in part due to the infrastructure and organisational arrangements that did not ensure patient privacy. Such views have been documented also in other settings [12,14]. This can be conceptualized as a form of structural or institutional stigma [18]. Ways of organising the services so that they do not add to or reinforce the stigma constraining people's testing decisions have been proposed, and studies in Zambia and other African countries have demonstrated that when VCT services have been made more convenient, confidential and locally acceptable through home-based or mobile services, uptake has increased substantially [12,51-57]. These studies indicate that the HIV related stigma may not remain an insurmountable barrier to HIV testing, and add importantly to debates concerning HIV testing approaches. In a context where the implementation of opt-out testing approaches has been criticized for compromising preventive counselling and processes of consent [22-24], the above studies provide hope that opt-in approaches with high standards of consent and counselling may yield high test rates if one avoids public exposure experienced at public testing sites.

The HIV epidemic is characterized by a shifting landscape of stigma, and the increasing availability of ART is hoped and expected to make an impact in the long run. There is a possibility that the scenario may have changed since these data were collected in 2007. Some of the informants had not experienced the anticipated stigma after being diagnosed with HIV, which may be an indication of processes of change. In the present study there was a relatively low representation of young people. As the motivation to test may be somewhat different among young people [56], certain dimensions of people's testing decisions may lack in the analysis, and further research is required in this area. However, young people did express the most negative views on receiving an HIV diagnosis, and we therefore do not think that including more young people would have given a substantially different picture of what is presented here. In our study we only employed one focus group discussion at the end of the field work in each area. Prior to the study we anticipated that HIV testing decisions and experiences would be sensitive issues, and therefore that the privacy offered in individual in-depth interviews would be more conducive. Contrary to our expectations, the interaction in the focus group discussions yielded a very rich material. If we were to do the study again, more focus group discussions would have been included in the study design.

## Conclusions

The present study revealed how decisions of whether or not to be tested for HIV are embedded in social and interpersonal processes constraining people's choices. The strongly promoted message 'to know your status' proved to be a highly charged concept yielding strong barriers against HIV testing. The study revealed that VCT was perceived as a diagnostic device and a gateway to treatment for the severely ill. Known benefits concerning prevention and early treatment were outweighed by a perceived burden of knowing one's HIV status. The manner in which VCT services were organised was found to strongly add to this burden. The burden of knowing and the related reluctance to get HIV tested can be understood both as a form of label-avoidance and as strong expressions of the still powerful embodied memories of suffering and death among non-curable AIDS patients over the last decades The meanings constructed around HIV testing in the present study are not entirely new. However, the findings are nonetheless of importance as meaning always emerges in slightly different forms, and will differ somewhat in content between people, time and places. Documenting such differences through locally grounded knowledge provides insights into the complexities of why client-initiated HIV test rates remain low, and may improve our thinking about ways ahead in the field of HIV testing. Hope lies in the emerging signs of a reduction in HIV related stigma experienced by those who had been tested and where ART is presently available. Taking seriously how meaning surfaces in particular times and places remains vital when we seek to increase our chances of finding "the difference that makes the difference" [58] in diverse HIV testing contexts.

## Competing interests

The authors declare that they have no competing interests.

## Authors' contributions

MJ and AB designed the study. MJ and MT conducted the interviews and focus group discussions. MJ analysed the data and drafted the manuscript. MT, KF and AB contributed to the interpretation of findings and revising the manuscript. AB and KF supervised the study and contributed in the literature review. All authors read and approved the final manuscript.

## Pre-publication history

The pre-publication history for this paper can be accessed here:

http://www.biomedcentral.com/1472-6963/12/2/prepub
